# Feasibility of repetitive transcranial magnetic stimulation on non-motor symptoms of spinocerebellar ataxia type 3: a secondary analysis of a randomized clinical trial

**DOI:** 10.3389/fneur.2025.1567292

**Published:** 2025-05-23

**Authors:** Hua Wu, Hao-Ling Xu, Xia-Hua Liu, Arif Sikandar, Wei Lin, Mao-Lin Cui, Ming-Xia Kang, Yi-Ru Zheng, Shi-Rui Gan, Liang-Liang Qiu

**Affiliations:** ^1^Department of Neurology and Institute of Neurology, The First Affiliated Hospital, Fujian Medical University, Fuzhou, China; ^2^Department of Neurology, National Regional Medical Center, Binhai Campus of the First Affiliated Hospital, Fujian Medical University, Fuzhou, China; ^3^Department of Rehabilitation Medicine, The First Affiliated Hospital, Fujian Medical University, Fuzhou, China

**Keywords:** repetitive transcranial magnetic stimulation (rTMS), spinocerebellar ataxia type 3 (SCA3), non-motor symptoms (NMS), sleep disturbance, cerebellum, cognitive deficits, mood disturbances

## Abstract

**Introduction:**

Spinocerebellar ataxia type 3 (SCA3) poses challenges for patients due to motor dysfunctions and non-motor symptoms (NMS), such as sleep disorders, cognitive deficits, and mood disturbances. These issues significantly impact the quality of life, with limited benefits from conventional pharmacotherapies. This study explores the potential of repetitive transcranial magnetic stimulation (rTMS) as a treatment for SCA3-related NMS.

**Methods:**

This is a secondary analysis of a randomized, double-blind, sham-controlled trial (The Chinese Clinical Trial Registry identifier is ChiCTR1800020133). Thirty-seven SCA3 patients included underwent either real (*n* = 21) or sham (*n* = 16) rTMS over 15 days, targeting the cerebellum. Study outcomes included the Pittsburgh Sleep Quality Index (PSQI), Athens Insomnia Scale (AIS), Mini-Mental State Examination (MMSE), Montreal Cognitive Assessment (MoCA), Hamilton Anxiety Rating Scale (HARS), and Hamilton Depression Rating Scale (HDRS), assessed baseline and post-intervention.

**Results:**

The results disclosed significant time effects for all the outcomes with *post hoc* comparisons showing differences of baseline and post-treatment evaluation, with decreases for PSQI, AIS, HARS, and HDRS as well as increase for MMSE and MoCA. Correlation analyses revealed no significant predictors of rTMS response based on age at onset, disease duration, number of expanded CAG repeat lengths, or baseline motor symptom severity scores.

**Conclusion:**

Repetitive transcranial magnetic stimulation is a feasible treatment of non-motor related symptoms in patients with SCA3, including sleep, cognition, and mood disorders. The treatment is well-tolerated, and its feasibility appears independent of demographic or disease severity indicators. These findings encourage further exploration of rTMS as a safe alternative for managing SCA3 NMS.

## Introduction

Spinocerebellar ataxia type 3 (SCA3), also known as Machado-Joseph disease, manifests as a progressive neurodegenerative disorder characterized by motor dysfunctions, including ataxia, dysarthria, and oculomotor abnormalities. As the most prevalent subtype of autosomal dominant ataxias, SCA3 stems from the expansion of cytosine-adenine-guanine (CAG) repeats in the *ATXN3* gene, resulting in a toxic gain of function primarily affecting the cerebellum and its associated pathways (1). While motor symptoms are well-recognized, non-motor symptoms (NMS) such as sleep disorders, cognitive deficits, and mood disturbances are also prevalent in SCA3. The frequency and severity of these NMS increase with the progressive worsening of ataxic severity, which is likely mediated by the cerebellum’s extensive connectivity with supratentorial networks. As cerebellar degeneration advances (evident in motor dysfunction), disruptions in cerebello-thalamo-cortical and cerebello-limbic pathways exacerbate NMS (2, 3). These symptoms significantly deteriorate the quality of life for patients, imposing a substantial economic and social burden on families (4). Despite the prevalence of NMS in these patients, few clinical trials specifically target these issues in SCA3.

Conventional pharmacotherapy provides some relief for NMS such as insomnia and mood disorders, utilizing agents like mirtazapine, melatonin, or anxiolytics and antidepressants. However, their use is limited by a high risk of adverse effects (e.g., sedation, dependency) and minimal efficacy for cognitive impairments (5). While non-pharmacological approaches like physiotherapy and transcranial direct current stimulation (6) have been explored for motor symptoms in SCA3 (6, 7), evidence for NMS remains scarce. In particular, no validated interventions exist for SCA3-related cognitive deficits, which are associated with cerebellar volume loss (7, 8). Hence, there is a pressing need to explore alternative therapies that address the multi-domain NMS spectrum—sleep, cognition, and mood—while minimizing side effects.

Repetitive transcranial magnetic stimulation (rTMS), a non-invasive procedure employing magnetic fields to stimulate nerve cells in the brain, has shown promise in various neurological and psychiatric disorders, including depression, anxiety disorders, Parkinson’s disease, and stroke (9). In the context of SCA3, prior randomized controlled trials (RCTs) including our team’s study (registered under ChiCTR1800020133) have demonstrated that cerebellar rTMS alleviates motor symptoms, particularly ataxia (10, 11). Mechanistically, low-frequency rTMS (1 Hz) may restore inhibitory control over hyperexcitable cerebellar circuits in SCA3 while enhancing dopaminergic and serotonergic transmission in limbic regions (12, 13). These findings position rTMS as both a safe motor intervention and a promising modality for addressing NMS through its dual action on cerebellar excitability and cortico-limbic connectivity.

In response to the increasing recognition of NMS in SCA3 and the demand for effective treatments, we conducted this secondary analysis based on our previous RCT study. This analysis clarifies that cerebellar rTMS is feasible for treating non-motor symptoms of SCA3, accompanied by non-significant side effects.

## Methods

### Study design and settings

The current study was a prespecified secondary analysis of our prior RCT (ChiCTR1800020133), a prospective, randomized, double-blind, sham-controlled study conducted between December 2018 and May 2019 at The First Affiliated Hospital of Fujian Medical University in Fuzhou, China. This trial’s design and primary results have been previously reported (10). We aimed to further investigate whether cerebellar rTMS would be feasible for treating NMS in SCA3 patients. This study received approval from the ethics committee of the First Affiliated Hospital of Fujian Medical University (ethics approval number: MRCTA, ECFAH of FMU [2018] 201). Patients in the current study were requested to provide separate informed consent for participation in a subgroup study focusing on non-motor symptoms.

### Participants

This secondary analysis utilized the parent study’s enrollment criteria without NMS-specific screening (10). However, as shown in Table 1, all participants demonstrated measurable NMS at baseline. The number of CAG repeats in the normal and mutant alleles of the *ATXN3* gene was determined using the polymerase chain reaction (PCR) technique, with subsequent validation of results through Sanger sequencing, in accordance with methodologies established in prior research (14).

**Table 1:** Sample characteristics at baseline.

Variables	Real rTMS group	Sham rTMS group	*p*-value
Total *n*	21	16	NA
Female sex, *n* (%)	11 (52.4%)	9 (56.3%)	0.82^b^
Education, median (range), y	‑9 (0, 16)	9 (4, 16)	0.37^c^
Age at enrolment, mean (SD), y	43.8 (11.4)	43.8 (11.8)	0.99^d^
Age at onset, mean (SD), y	35.3 (11.5)	34.0 (10.1)^a^	0.72^d^
Disease duration, mean (SD), y	10.1 (3.9)	9.3 (5.5)^a^	0.62^d^
Number of normal CAG repeats, median (range)	14 (14, 30)	14 (14, 44)	0.99^c^
Number of expanded CAG repeats, mean (SD)	75.1 (3.6)	75.7 (3.4)	0.59^d^
SARA scores, mean (SD), points	15.6 (6.7)	12.8 (5.05)	0.18^d^
ICARS scores, mean (SD), points	40.6 (16.2)	33.6 (14.4)	0.18^d^

SARA, Scale for the Assessment and Rating of Ataxia; ICARS, International Cooperative Ataxia Rating Scale; rTMS, repetitive transcranial magnetic stimulation; NA, not applicable.

^a^One patient did not report her age at onset.

^b^*χ*^2^ test.

^c^Mann–Whitney *U* test.

^d^Independent samples *t*-test. The bold values meaning *p*-value < 0.05.

### Intervention

Participants received 15 daily sessions of cerebellar rTMS (1 Hz, 100% RMT, 900 pulses/hemisphere) using a CCY-I stimulator (Yiruide, China). Real stimulation was delivered tangentially with a 14 cm circular coil positioned 4 cm lateral to the inion bilaterally, while sham used vertical coil placement. Patients were instructed to discontinue sleep medication 3 days before the start of stimulation and to remain without medication during rTMS therapy. Documented side effects from rTMS treatment include nausea, localized pain at the stimulation site, discomfort in the neck, muscular rigidity in the cervical region, headaches, manifestations of psychosis, seizures, and temporary alterations in auditory perception (15, 16).

### Measurement

Baseline characteristics, including demographics, age at onset, disease duration, motor function, and genetic information, were obtained. Motor function evaluations included scores of the Scale for the Assessment and Rating of Ataxia (SARA) (17) and the International Cooperative Ataxia Rating Scale (ICARS) (18).

Integrated evaluations of NMS were performed at baseline (day 1) and post-treatment (day 15), including sleep quality, cognitive function, and emotional well-being. Trained personnel of Dr. Hua Wu, who were blinded to both intervention arms, conducted evaluations to ensure standardization and reliability.

Sleep-related indices were measured by the Pittsburgh Sleep Quality Index (PSQI) (19) and the Athens Insomnia Scale (AIS) (20). The PSQI, a tool for evaluating sleep quality and disturbances, comprises 19 items forming seven component scores reflecting various aspects of sleep over the past month. Each component is equally weighted on a 0–3 scale, with the sum producing a global score ranging from 0 to 21, where lower scores indicate better sleep quality. Conversely, the AIS focuses on the severity of insomnia symptoms, with eight items covering sleep induction, nocturnal awakenings, and daytime functioning related to sleep quality. Each item is scored from 0 (no problem) to 3 (very serious problem), and the total score ranges from 0 to 24, with higher scores indicating more severe insomnia. Cognitive indices were measured by the Mini-Mental State Examination (MMSE) (21) and the Montreal Cognitive Assessment (MoCA) (22). The MMSE, a widely used and validated tool for screening cognitive impairment, consists of simple questions and problems in areas such as arithmetic, memory, and orientation, with a total possible score of 30 points. Lower MMSE scores indicate more severe cognitive impairment. The MoCA, a more recent cognitive screening tool, assesses a broader spectrum of cognitive functions, including executive functions, attention, concentration, working memory, language, visuo-constructional skills, conceptual thinking, calculations, and orientation. It has a maximum score of 30 points, with higher scores indicating better cognitive function. Psychometric assessments were measured by the Hamilton Anxiety Rating Scale (HARS) (23) and the Hamilton Depression Rating Scale (HDRS) (24). The HARS and HDRS are established rating scales designed to measure the severity of anxiety and depression symptoms, respectively, and are crucial for evaluating the psychological dimensions associated with sleep disorders. Higher scores on HARS and HDRS indicate greater severity of anxiety and depression symptoms.

### Study outcomes

The primary outcomes of this analysis were sleep-related indices including scores of PSQI and AIS at post-treatment (day 15). The secondary outcomes of this analysis encompassed cognitive indices, including scores of MMSE and MoCA, and psychometric assessments, including scores of HARS and HDRS, at post-treatment (day 15).

### Statistical analysis

This secondary analysis was exploratory. Sample size was determined by the parent RCT (10), which was calculated using an effect size of 0.92 (partial *η*^2^ = 0.46 from prior data), with *α* = 0.05 and 80% power for ICARS as the primary outcome.

For the primary analysis, generalized estimating equation (GEE) models were used to compare outcomes between the real rTMS and sham rTMS groups (between-subjects) and between baseline and day 15 assessments (within-subjects). Each study outcome served as the dependent variable, with the experimental group as the categorical predictor. Mean differences (MDs) with 95% confidence intervals (CI) were calculated.

Continuous parametric data are reported as the mean ± standard deviations (SDs), while non-parametric data are presented as the median (range). Categorical variables are denoted as frequencies (%). The Shapiro–Wilk test assessed normality for all variables. The Mann–Whitney *U* test was used to analyze non-parametric continuous variables across two groups, while independent samples *t*-test was used to analyzed normally distributed variables. Categorical variables were analyzed using the *χ*^2^ test or Fisher’s exact test in cases where expected frequencies were less than 5. The Spearman rank correlation coefficient (for non-normal data) or Pearson’s correlation analysis (for normal data) explored the relationships between variables.

All analyses were performed in SPSS (v20.0; IBM Corp., Armonk, NY, United States). The level of statistical significance was set at *p* less than 0.05.

## Results

**Figure 1:**
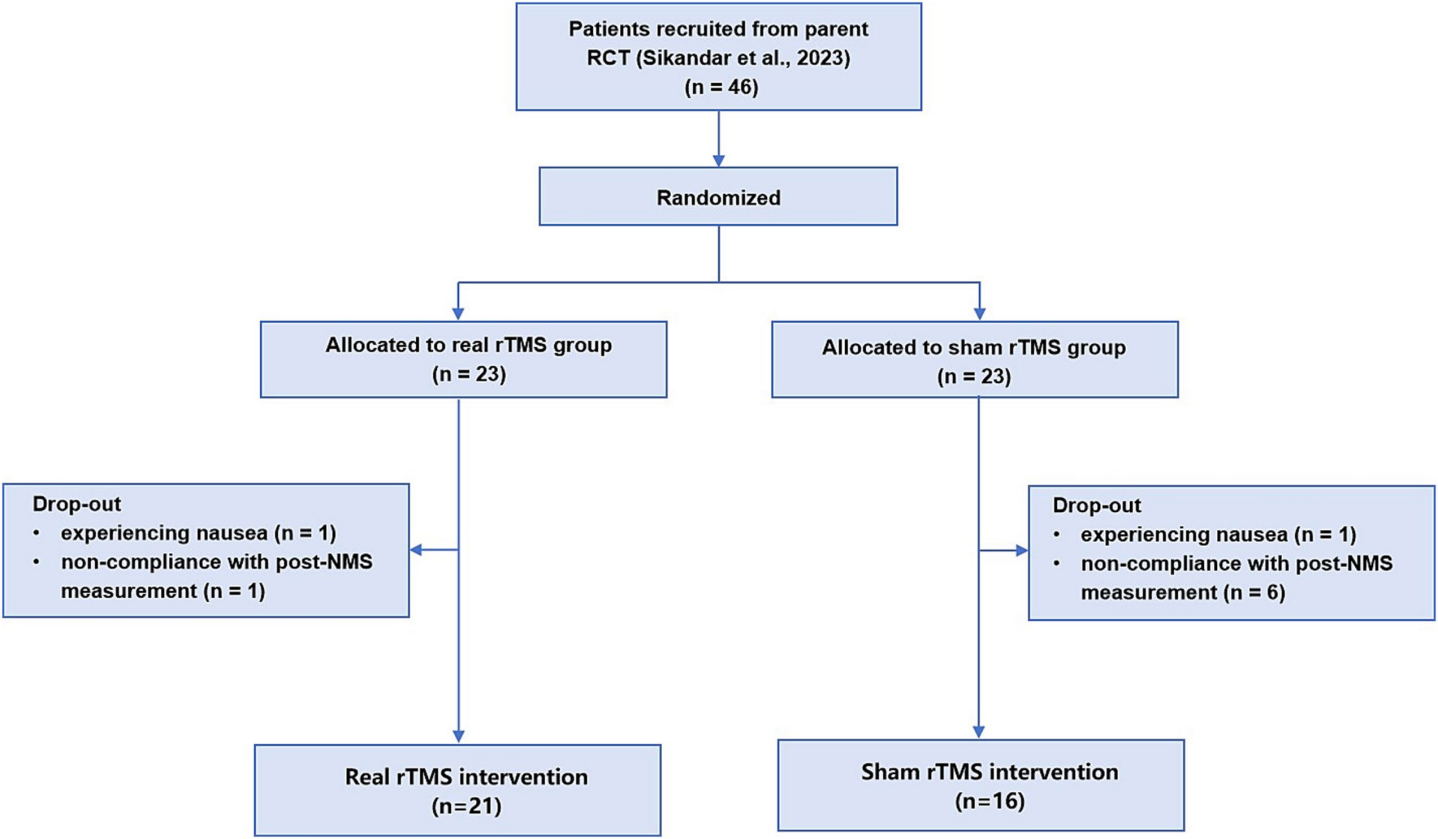
The flow chart.

As shown Figure 1 in the 46 patients in our prior RCT (ChiCTR1800020133), who completed NMS and motor measurements, were allocated 1:1 into the real or sham rTMS group. Nine patients were excluded in this secondary analysis: two patients due to experiencing slight side effect of nausea and seven patients due to non-compliance with post-NMS measurement. Ultimately, 37 patients were included in this analysis (median age, 45 years [IQR, 35-52 years]; 20 [54.1%] female), 21 patients in the real rTMS group and 16 patients in the sham group (Figure 1).

### Baseline characteristics

Comparisons of baseline characteristics between the two groups of real and sham group revealed no significant disparities in sex distribution (*p* = 0.82), year of education (*p* = 0.37), age at enrollment (*p* = 0.99), age at onset (*p* = 0.72), disease duration (*p* = 0.62), or the number of expanded CAG repeats (*p* = 0.59). The SARA (*p* = 0.18) and ICARS (*p* = 0.18) scores were also comparable (Table 1). Individual details can be found in the Supplementary Table 1. Therefore, baseline disease-related variables between the two groups were expected to be equal at the beginning of the analysis.

### Study outcomes

The GEE disclosed significant time effects for all the outcomes of PSQI (MD, −3.73, [95%CI, –4.92 –2.54];, *p* < 0.001), AIS (MD, −3.41, [95%CI, –4.97 –1.84];, *p* < 0.001), MMSE (MD, 0.73, [95%CI, 0.26–1.2];, *p* = 0.002), MoCA (MD, 2.41, [95%CI, 1.63–3.18];, *p* < 0.001), HARS (MD, −7.49, [95%CI, –10.02 –4.95];, *p* = 0.01), and HDRS (MD, −5.35, [95%CI, –7.21–3.49];, *p* < 0.001), with *post hoc* comparisons showing differences of baseline and post-treatment evaluation. In addition, no significant group effects or interaction effects (stimulation [baseline-post] ╳ group [real-sham]) was found either at the baseline or 15-day assessments. The results of outcomes were summarized in Table 2. Moreover, mean comparisons between baseline and 15-day measures within the real group disclosed significant decreases for PSQI, AIS, HARS, and HDRS as well as disclosed significant increases for MMSE and MoCA. Results within the sham group also found decreases for PSQI, AIS, HARS, and HDRS as well as found increases for MoCA (Supplementary Table 2).

**Table 2:** Study outcomes.

Variables	Real rTMS group		Sham rTMS group		GEE findings	
	Baseline	Day 15	Baseline	Day 15	Mean Difference (95%CI)	*p*-value^a^
PSQI	11.6 (5.1)	7.4 (4.0)	9.6 (4.9)	6.5 (3.4)	−3.73 (−4.92, −2.54)	**<0.001**
AIS	9.5 (5.4)	5.7 (3.7)	8.9 (6.1)	6.1 (2.7)	−3.41 (−4.97, −1.84)	**<0.001**
MMSE	26.3 (3.0)	27.6 (2.2)	27.8 (2.4)	27.9 (1.4)	0.73 (0.26, 1.2)	**0.002**
MoCA	21.3 (5.9)	23.6 (5.8)	21.8 (4.0)	24.4 (4.6)	2.41 (1.63, 3.18)	**<0.001**
HARS	21.9 (7.4)	13.8 (8.1)	21.3 (9.7)	14.5 (9.0)	−7.49 (−10.02, −4.95)	**0.01**
HDRS	21.6 (6.0)	15.6 (6.0)	20.8 (10.6)	16.3 (8.2)	−5.35 (−7.21, 3.49)	**<0.001**

PSQI, the Pittsburgh Sleep Quality Index; AIS, Athens Insomnia Scale; MMSE, Mini-Mental State Examination; MoCA, Montreal Cognitive Assessment; HARS, Hamilton Anxiety Rating Scale; HDRS, Hamilton Depression Rating Scale; rTMS, repetitive transcranial magnetic stimulation; GEE, Generalized Estimating Equations.

Values are expressed as mean (standard deviation [SD]).

^a^Time effects.The bold values meaning *p*-value < 0.05.

No significant correlations were observed between treatment response (ΔPSQI, ΔAIS, ΔMMSE, ΔMoCA, ΔHARS, and ΔHDRS) and any of the investigated predictors (age at onset, disease duration, the number of expanded CAG repeats, or baseline motor symptom severity scores [SARA/ICARS]) in the real stimulation group (all *p* > 0.05; Table 3).

**Table 3:** The investigation of the predictors in the effect of rTMS.

Variables	ΔPSQI		ΔAIS		ΔMMSE		ΔMoCA		ΔHARS		ΔHDRS	
	*r*/*ρ*^a^	*p*-value	*r*/*ρ*^b^	*p*-value	*r*/*ρ*^a^	*p*-value	*r*/*ρ*^b^	*p*-value	*r*/*ρ*^b^	*p*-value	*r*/*ρ*^b^	*p*-value
Age at onset, y	−0.17	0.47	0.11	0.63	0.03	0.89	0.04	0.86	0.31	0.16	0.30	0.19
Disease duration, y	0.05	0.84	0.36	0.11	0.16	0.48	−0.003	0.99	0.32	0.16	0.36	0.11
Number of expanded CAG repeats	0.34	0.13	0.10	0.68	0.04	0.87	−0.03	0.91	−0.01	0.98	−0.06	0.79
Baseline SARA scores, points	−0.11	0.63	0.09	0.70	−0.17	0.45	−0.29	0.91	0.22	0.34	0.15	0.51
Baseline ICARS scores, points	−0.06	0.80	0.08	0.74	−0.15	0.52	−0.28	0.22	0.22	0.35	0.13	0.59

SARA, Scale for the Assessment and Rating of Ataxia; ICARS, International Cooperative Ataxia Rating Scale; PSQI, the Pittsburgh Sleep Quality Index; AIS, Athens Insomnia Scale; HARS, Hamilton Anxiety Rating Scale; HDRS, Hamilton Depression Rating Scale.

ΔPSQI was non-normal data, all the other variables were normal data.

^a^Spearman rank correlation coefficient (*ρ*).

^b^Pearson’s correlation analysis (*r*).

Apart from two patients who experienced slight nausea, no other significant adverse reactions were observed in the other patients, indicating good tolerance for both the real and sham rTMS interventions.

## Discussion

This present secondary analysis reported a feasible treatment for NMS in SCA3, an area historically underrepresented in clinical research. Our findings suggested that cerebellar rTMS was a feasible and non-invasive therapeutic option for SCA3 patients experiencing sleep disorders, cognitive deficits, and mood disturbances.

This study underscored the cerebellum’s expanded role beyond motor function, highlighting its crucial involvement in broader neurological processes, including NMS (2, 3). The cerebellum’s extensive connections with the cerebral cortex and limbic system position it as a pivotal node within networks regulating affective and cognitive functions. Disruptions in cerebellar output can dysregulate these networks, contributing to the observed NMS in SCA3 (25). While the precise mechanisms linking cerebellar dysfunction to NMS remain unclear, they may encompass altered neurotransmission, synaptic plasticity, and connectivity with other brain regions (26). Targeting the cerebellum, rTMS holds promise in restoring the balance of excitatory and inhibitory inputs, normalizing disrupted circuits, and promoting neuroplastic changes.

Patients receiving cerebellar rTMS after 15 consecutive days showed improvements in their PSQI and AIS scores in our analysis. These outcomes can be attributed to the modulatory effects of rTMS on neural networks, neuroplasticity, and neurotransmitter release involved in sleep regulation (13, 27). On the other hand, previous studies have shown that cognitive impairment was one of the major NMS of SCA3 (2), and it was associated with cerebellar volume reduction (28). Our results suggested that cerebellar rTMS was beneficial for addressing cognitive impairment (increasing in MMSE and MoCA scores). Additionally, the rTMS intervention exhibited reductions in both HARS and HDRS scores. This aligned with prior research demonstrating the feasibility of rTMS in treating primary mood disorders, such as anxiety and depression (29, 30). In the context of SCA3, rTMS may exert antidepressant and anxiolytic effects by altering cerebellar connectivity with limbic structures, such as the hippocampus and amygdala, known to regulate mood and anxiety (31). The therapeutic benefits observed for sleep quality, cognition, and mood in our study were particularly compelling, given the relatively low risk of adverse effects associated with rTMS compared to pharmacological treatments.

While this secondary analysis revealed improvements in NMS following cerebellar rTMS, the lack of group or interaction effects suggests both groups improved, likely due to two possibilities: First, placebo effects and/or non-specific stimulation, this aligns with findings from our primary RCT, where sham groups also showed transient motor improvements (10). Second, MMSE and MoCA screeners lack sensitivity for cerebellar-mediated cognitive deficits (e.g., executive dysfunction, processing speed) as highlighted in recent systematic reviews (32). Our choice was constrained by parent study design prioritizing motor outcome and limiting time for detailed neuropsychological testing. Future trials should incorporate domain-specific Cerebellar Cognitive Affective Syndrome (CCAS) scales (32, 33). The lack of correlation between treatment response and disease-related variables, such as age at onset, disease duration, baseline motor symptom severity, and the number of expanded CAG repeat length, implied that rTMS may have broad applicability across the SCA3 population. The absence of significant correlations between these variables and the therapeutic benefits of rTMS could be attributed to several factors. Firstly, the sample size of our study might not have been sufficient to detect subtle correlations. Future studies incorporating cerebellar-specific neuropsychological tests may offer a more comprehensive understanding of the relationships between these variables and the therapeutic response to rTMS. Secondly, while genetically homogeneous, our cohort exhibited marked clinical heterogeneity in motor and non-motor manifestations—a hallmark of polyglutamine disorders that must be accounted for in therapeutic trials (1, 34). It was worth noting that our study included a relatively homogenous population with similar baseline characteristics, which might limit the generalizability of our findings to more diverse patient populations.

The dropout rate for this study was relatively low. Two participants discontinued their participation due to experiencing slightly adverse reaction of nausea, with one from the real stimulation group and one from the sham stimulation group. An additional seven participants withdrew because they were unwilling to comply with the post-intervention assessment scales. This suggested that the intervention was generally well-tolerated by the participants, with no significant side effects reported. These findings aligned with previous studies demonstrating the safety and tolerability of rTMS in various neurological disorders.

While this study provided valuable insights, it was important to note a few limitations. Firstly, being a secondary analysis, there was an elevated risk of Type I errors due to multiple testing. Therefore, the results should be considered with caution. Secondly, our analysis focused on assessing the immediate effects of rTMS. A study with a larger sample size and a longer follow-up period could provide more insights into the enduring benefits of low-frequency rTMS for individuals with SCA3. Thirdly, the lack of formal NMS inclusion thresholds, while reflecting real-world clinical heterogeneity, may have introduced variability in treatment responses. Lastly, the 3-day medication washout, while balancing patient safety and scientific rigor, may not fully eliminate all pharmacological effects. Future primary studies should consider longer washouts where clinically feasible.

## Conclusion

In summary, this study contributed to the growing body of evidence supporting the use of rTMS for non-motor symptoms in SCA3. The demonstrated improvements in sleep quality, cognitive function, and mood disorders without significant side effects suggested that rTMS could be a feasible and valuable addition to the therapeutic options for SCA3. Future research should focus on optimizing stimulation parameters and cerebellar lobes, exploring potential combination therapies, and investigating the long-term outcomes of rTMS treatment. By enhancing our understanding of the non-motor symptoms of SCA3 and the therapeutic potential of neuromodulation, we can progress toward providing comprehensive care that addresses the entire spectrum of patient needs.

## Data Availability

The original contributions presented in the study are included in the article/Supplementary material, further inquiries can be directed to the corresponding authors.

## References

[1] Klockgether T Mariotti C Paulson HL. Spinocerebellar ataxia. Nat Rev Dis Primers. (2019) 5:24. doi: 10.1038/s41572-019-0074-3, PMID: 30975995

[2] Hengel H Martus P Faber J Giunit P Garcia-Moreno H Solanky N. The frequency of non-motor symptoms in SCA3 and their association with disease severity and lifestyle factors. J Neurol. (2023) 270:944–52. doi: 10.1007/s00415-022-11441-z, PMID: 36324033 PMC9886646

[3] Pedroso JL França MC Jr Braga-Neto P D'Abreu A Saraiva-Pereira ML Saute JA. Non-motor and extracerebellar features in Machado-Joseph disease: a review. Mov Disord. (2013) 28:1200–8. doi: 10.1002/mds.25513, PMID: 23775899

[4] Schmitz-Hübsch T Coudert M Giunti P Globas C Baliko L Fancellu R. Self-rated health status in spinocerebellar ataxia--results from a European multicenter study. Mov Disord. (2010) 25:587–95. doi: 10.1002/mds.22740, PMID: 20175183

[5] Oliveira JBL Martinez ARM França MC Jr. Pharmacotherapy for the management of the symptoms of Machado-Joseph disease. Expert Opin Pharmacother. (2022) 23:1687–94. doi: 10.1080/14656566.2022.2135432, PMID: 36254604

[6] Maas R Teerenstra S Toni I Klockgether T Schutter DJLG van de Warrenburg B. Cerebellar transcranial direct current stimulation in spinocerebellar Ataxia type 3: a randomized, double-blind, sham-controlled trial. Neurotherapeutics. (2022) 19:1259–72. doi: 10.1007/s13311-022-01231-w, PMID: 35501469 PMC9059914

[7] Yap KH Azmin S Che Hamzah J Ahmad N van de Warrenburg B Mohamed Ibrahim N. Pharmacological and non-pharmacological management of spinocerebellar ataxia: a systematic review. J Neurol. (2022) 269:2315–37. doi: 10.1007/s00415-021-10874-2, PMID: 34743220

[8] Liu H Lin J Shang H. Voxel-based meta-analysis of gray matter and white matter changes in patients with spinocerebellar ataxia type 3. Front Neurol. (2023) 14:1197822. doi: 10.3389/fneur.2023.1197822, PMID: 37576018 PMC10413272

[9] Qiu M Wang R Shen Y Hu Z Zhang Y. Efficacy and safety of repetitive transcranial magnetic stimulation in spinocerebellar Ataxia type 3: a systematic review and meta-analysis of randomized controlled trials. Cerebellum. (2023) 23:1604–13. doi: 10.1007/s12311-023-01628-z, PMID: 37975968

[10] Sikandar A Liu XH Xu HL Li Y Lin YQ Chen XY. Short-term efficacy of repetitive transcranial magnetic stimulation in SCA3: a prospective, randomized, double-blind, sham-controlled study. Parkinsonism Relat Disord. (2023) 106:105236. doi: 10.1016/j.parkreldis.2022.105236, PMID: 36529111

[11] França C de Andrade DC Silva V Galhardoni R Barbosa ER Teixeira MJ. Effects of cerebellar transcranial magnetic stimulation on ataxias: a randomized trial. Parkinsonism Relat Disord. (2020) 80:1–6. doi: 10.1016/j.parkreldis.2020.09.001, PMID: 32920321

[12] Rodríguez-Labrada R Velázquez-Pérez L Ziemann U. Transcranial magnetic stimulation in hereditary ataxias: diagnostic utility, pathophysiological insight and treatment. Clin Neurophysiol. (2018) 129:1688–98. doi: 10.1016/j.clinph.2018.06.003, PMID: 29940480

[13] Lefaucheur JP Aleman A Baeken C Benninger DH Brunelin J di Lazzaro V. Evidence-based guidelines on the therapeutic use of repetitive transcranial magnetic stimulation (rTMS): an update (2014-2018). Clin Neurophysiol. (2020) 131:474–528. doi: 10.1016/j.clinph.2019.11.002, PMID: 31901449

[14] Gan SR Ni W Dong Y Wang N Wu ZY. Population genetics and new insight into range of CAG repeats of spinocerebellar ataxia type 3 in the Han Chinese population. PLoS One. (2015) 10:e0134405. doi: 10.1371/journal.pone.0134405, PMID: 26266536 PMC4534407

[15] Abler B Walter H Wunderlich A Grothe J Schönfeldt-Lecuona C Spitzer M. Side effects of transcranial magnetic stimulation biased task performance in a cognitive neuroscience study. Brain Topogr. (2005) 17:193–6. doi: 10.1007/s10548-005-6028-y, PMID: 16110769

[16] Rossi S Antal A Bestmann S Bikson M Brewer C Brockmöller J. Safety and recommendations for TMS use in healthy subjects and patient populations, with updates on training, ethical and regulatory issues: expert guidelines. Clin Neurophysiol. (2021) 132:269–306. doi: 10.1016/j.clinph.2020.10.003, PMID: 33243615 PMC9094636

[17] Schmitz-Hübsch T du Montcel ST Baliko L Berciano J Boesch S Depondt C. Scale for the assessment and rating of ataxia: development of a new clinical scale. Neurology. (2006) 66:1717–20. doi: 10.1212/01.wnl.0000219042.60538.92, PMID: 16769946

[18] Trouillas P Takayanagi T Hallett M Currier RD Subramony SH Wessel K. International cooperative Ataxia rating scale for pharmacological assessment of the cerebellar syndrome. The Ataxia neuropharmacology Committee of the World Federation of neurology. J Neurol Sci. (1997) 145:205–11. doi: 10.1016/S0022-510X(96)00231-6, PMID: 9094050

[19] Buysse DJ Reynolds CF Monk TH Berman SR Kupfer DJ. The Pittsburgh sleep quality index: a new instrument for psychiatric practice and research. Psychiatry Res. (1989) 28:193–213. doi: 10.1016/0165-1781(89)90047-4, PMID: 2748771

[20] Soldatos CR Dikeos DG Paparrigopoulos TJ. Athens insomnia scale: validation of an instrument based on ICD-10 criteria. J Psychosom Res. (2000) 48:555–60. doi: 10.1016/S0022-3999(00)00095-7, PMID: 11033374

[21] Folstein MF Folstein SE McHugh PR. “Mini-mental state”. A practical method for grading the cognitive state of patients for the clinician. J Psychiatr Res. (1975) 12:189–98. doi: 10.1016/0022-3956(75)90026-6, PMID: 1202204

[22] Nasreddine ZS Phillips NA Bédirian V Charbonneau S Whitehead V Collin I. The Montreal cognitive assessment, MoCA: a brief screening tool for mild cognitive impairment. J Am Geriatr Soc. (2005) 53:695–9. doi: 10.1111/j.1532-5415.2005.53221.x, PMID: 15817019

[23] Hamilton M. The assessment of anxiety states by rating. Br J Med Psychol. (1959) 32:50–5. doi: 10.1111/j.2044-8341.1959.tb00467.x, PMID: 13638508

[24] Hamilton M. A rating scale for depression. J Neurol Neurosurg Psychiatry. (1960) 23:56–62. doi: 10.1136/jnnp.23.1.56, PMID: 14399272 PMC495331

[25] Shinn AK Hurtado-Puerto AM Roh YS Ho V Hwang M Cohen BM. Cerebellar transcranial magnetic stimulation in psychotic disorders: intermittent, continuous, and sham theta-burst stimulation on time perception and symptom severity. Front Psych. (2023) 14:1218321. doi: 10.3389/fpsyt.2023.1218321, PMID: 38025437 PMC10679721

[26] Adamaszek M D’Agata F Ferrucci R Habas C Keulen S Kirkby KC. Consensus paper: cerebellum and emotion. Cerebellum. (2017) 16:552–76. doi: 10.1007/s12311-016-0815-8, PMID: 27485952

[27] Lanza G Fisicaro F Cantone M Pennisi M Cosentino FII Lanuzza B. Repetitive transcranial magnetic stimulation in primary sleep disorders. Sleep Med Rev. (2023) 67:101735. doi: 10.1016/j.smrv.2022.101735, PMID: 36563570

[28] Ye ZX Bi J Qiu LL Chen XY Li MC Chen XY. Cognitive impairment associated with cerebellar volume loss in spinocerebellar ataxia type 3. J Neurol. (2024) 271:918–28. doi: 10.1007/s00415-023-12042-0, PMID: 37848650

[29] Zhang L Zhu J Zhang T Jia Q Hui L Zhu H. Comparative efficacy of add-on rTMS in treating the somatic and psychic anxiety symptoms of depression comorbid with anxiety in adolescents, adults, and elderly patients-a real-world clinical application. J Affect Disord. (2020) 276:305–11. doi: 10.1016/j.jad.2020.05.151, PMID: 32871660

[30] Hutton TM Aaronson ST Carpenter LL Pages K West WS Kraemer C. The anxiolytic and antidepressant effects of transcranial magnetic stimulation in patients with anxious depression. J Clin Psychiatry. (2023) 84:4571. doi: 10.4088/JCP.22m14571, PMID: 36630648

[31] Schutter DJ van Honk J. The cerebellum on the rise in human emotion. Cerebellum. (2005) 4:290–4. doi: 10.1080/14734220500348584, PMID: 16321885

[32] Yap KH Kessels RPC Azmin S van de Warrenburg B Mohamed Ibrahim N. Neurocognitive changes in spinocerebellar Ataxia type 3: a systematic review with a narrative design. Cerebellum. (2022) 21:314–27. doi: 10.1007/s12311-021-01282-3, PMID: 34231180

[33] Maas RPPWM Killaars S van de Warrenburg BPC Schutter DJLG. The cerebellar cognitive affective syndrome scale reveals early neuropsychological deficits in SCA3 patients. J Neurol. (2021) 268:3456–66. doi: 10.1007/s00415-021-10516-7, PMID: 33743045 PMC8357713

[34] Du YC Dong Y Cheng HL Li QF Yang LS Hao YR. Genotype-phenotype correlation in 667 Chinese families with spinocerebellar ataxia type 3. Parkinsonism Relat Disord. (2020) 78:116–21. doi: 10.1016/j.parkreldis.2020.07.024, PMID: 32814229

